# Do alpha blockers reduce the risk of urinary retention post-transperineal prostate biopsy? A systematic narrative review

**DOI:** 10.1007/s00345-024-05001-5

**Published:** 2024-05-17

**Authors:** Zein Alhamdani, Samuel Poppenbeek, Damien Bolton, Lih-Ming Wong, Kapil Sethi

**Affiliations:** 1 Department of Urology, University of Melbourne, Austin Health, 145 Studley Rd, Heidelberg VIC, Melbourne, 3084 Australia; 2 Department of Surgery, University of Melbourne, St Vincent’s Health, Melbourne, Australia

**Keywords:** Transperineal Prostate biopsy, Urinary retention, Alpha blockers, Systematic review, Uro-oncology

## Abstract

**Background:**

Transperineal Prostate Biopsy (TPB) is a commonly used technique for the diagnosis of prostate cancer due to growing concerns related to infectious complications associated with transrectal ultrasound-guided prostate biopsy (TRUSB). TPB is associated with an infective complication rate of near zero, however, acute urinary retention (AUR) remains the leading complication causing morbidity. Previously in TRUSB, there was weak evidence that alpha-blockers reduce AUR rates, and their usage has been extrapolated to clinical practice with TPB. This review aims to explore if there is an evidence base for using alpha-blockers to prevent AUR following TPB.

**Methods:**

A systematic approach was used to search Ovid Medline and Embase using keywords related to “Transperineal” and “Retention”. Articles were then screened by applying inclusion and exclusion criteria to find studies that compared alpha-blocker recipients to no alpha-blocker use in the perioperative period and the subsequent effect on AUR in TPB.

**Results:**

361 records were identified in the initial search to produce 5 studies included in the final review. No randomised controlled trials (RCTs) were identified. One observational study showed a reduction in AUR rate from 12.5% to 5.3% with a single dose of tamsulosin. A previous systematic review of complications associated with prostate biopsy concluded there may be a potential benefit to alpha-blockers given in the TPB perioperative period. Three observational studies demonstrated a harmful effect related to alpha-blocker use; however, this was well explained by their clear limitations.

**Conclusion:**

Based on this review and the extrapolation from TRUSB data, perioperative alpha-blockers may offer some weak benefits in preventing AUR following TPB. However, there is significant scope and need for an RCT to further develop the evidence base further given the significant gap in the literature and lack of a standard alpha blocker protocol in TPB.

## Introduction

Prostate cancer is the most commonly diagnosed cancer in men across the world [1]. To diagnose prostate cancer, a biopsy of the suspected lesion is first required to histologically confirm the type of cancer and stage of disease. Previously, the most common method of biopsy was trans-rectal ultrasound guided biopsy (TRUSB), which involves the use of an ultrasound probe to visualise the prostate and facilitate a needle through the rectum to acquire the tissue samples. There is growing concern related to the infective complications associated with this technique, however, as the needle passes through the rectum into the prostatic tissues leading to sepsis in up to 6.3% of cases [2].

This trend has shifted the biopsy technique to favour the transperineal biopsy (TPB). This method allows the sterilisation of the perineum before needle access is obtained and has been shown to have a sepsis complication rate of near zero [3–5]. Other benefits of a transperineal approach compared to a transrectal include the ability to access the anterior prostate, resulting in a greater cancer detection rate [6]. The main complication associated with TPB however is now acute urinary retention (AUR). Previously, in TRUSB, there have been two clinical trials that have demonstrated a benefit to the use of perioperative Tamsulosin, a uroselective alpha-blocking medication, to prevent AUR complications [7, 8]. It is hypothesised that the needle sample taken during the biopsy leads to local tissue damage in the prostate. This causes oedema, swelling and bleeding or possible trauma to the urethra itself that then results in an outlet obstruction and AUR [2]. Alpha-blockers like Tamsulosin are hypothesised to relieve this [9]. This benefit has then been extrapolated to the TPB by some clinicians hoping to see the same advantage seen in the two RCTs on TRUSB [7, 8]. There is no clinical standard or established protocol for their usage, and not every clinician prescribes them as part of their practice when taking a TPB. This review sets out to examine if there is an evidence base for their use in TPB with a systematic approach, and examines the question, do perioperative alpha-blockers reduce the risk of AUR following TPB?

## Methods

### Selection criteria

#### Types of studies

Initially, the search criteria were restricted to only include randomised controlled trials (RCTs) comparing the benefit of perioperative alpha-blockers to a control on the reported urinary retention rates, however to the best of our knowledge there has been no RCT examining this in the setting of TPB. Given this gap in the literature, the search was widened to include retrospective studies, prospective cohort studies, national database series, and systematic reviews reporting alpha-blocker usage during TPB.

#### Types of participants

Papers were included if they examined men undergoing prostate biopsy via a transperineal approach. No limitations were placed on the specific technique used within the transperineal approach given the wide variety used in clinical practice.

#### Types of interventions and comparators

Studies were included if they commented on using of perioperative alpha-blockers and allowed a direct comparison to participants not taking alpha-blockers in the same period. The exact prophylactic regime of alpha-blocker use was not limited.

#### Types of outcome measures

Studies were included if they reported the AUR rates following TPB and could give a breakdown of the impact of an alpha-blocker in the perioperative period. In this review, AUR was defined by the need for temporary catheterisation between biopsy and follow-up review in the days/weeks after the biopsy. Additional information regarding further analysis of the risk factors for AUR following TPB was also sought; however, this was not part of the strict inclusion criteria concerning the aim of this study.

A summary of the inclusion/exclusion criteria can be found in Appendix 2.

### Search methods for identification of studies

#### Electronic searches

On 15th November 2023, two independent investigators (Z.A and S.P) utilised the electronic databases Embase and Ovid Medline® were searched around the key terms transperineal AND urinary retention along with their relevant MeSH headings combined with the OR Boolean operator. The entire search strategies can be found in Appendix 1.

### Data collection and extraction

#### Selection of studies

Using the online tool Covidence [10] which is a web-based collaboration software platform that streamlines the production of systematic reviews, the titles and abstracts of all studies identified as part of the search underwent initial screening. According to the inclusion and exclusion criteria, each study is marked as either yes, no, or unclear, with being marked as no resulting in exclusion from further evaluation. The papers marked yes or unclear then underwent full-text screening using the same inclusion and exclusion criteria to yield the final selection of studies. Any disputes in articles included or excluded were resolved by the use of a third author (K.S).

All studies included were assessed for their risk of bias utilising the Study Quality Assessment Tools provided by National Heart, Lung and Blood Institute [11] which assesses the rate of participation, justification for patient population and recruitment, time, exposure measures, outcome assessment, follow-up and adjustment for confounding factors. Each study was rated ‘good’, ‘fair’, or ‘poor’ according to the estimated risk of bias by individual assessment (Z.A and S.P), and any rating disagreements were resolved using a third author (K.S) to reach consensus.

#### Extraction of data

Using an excel template, the selected studies underwent data extraction. The type of study, aims, biopsy technique, anaesthetic type, number of biopsy cores, data collection methods, centre type, number of participants, breakdown of alpha-blocker use and AUR rates were collected.

## Results

### Search results

**Fig. 1:**
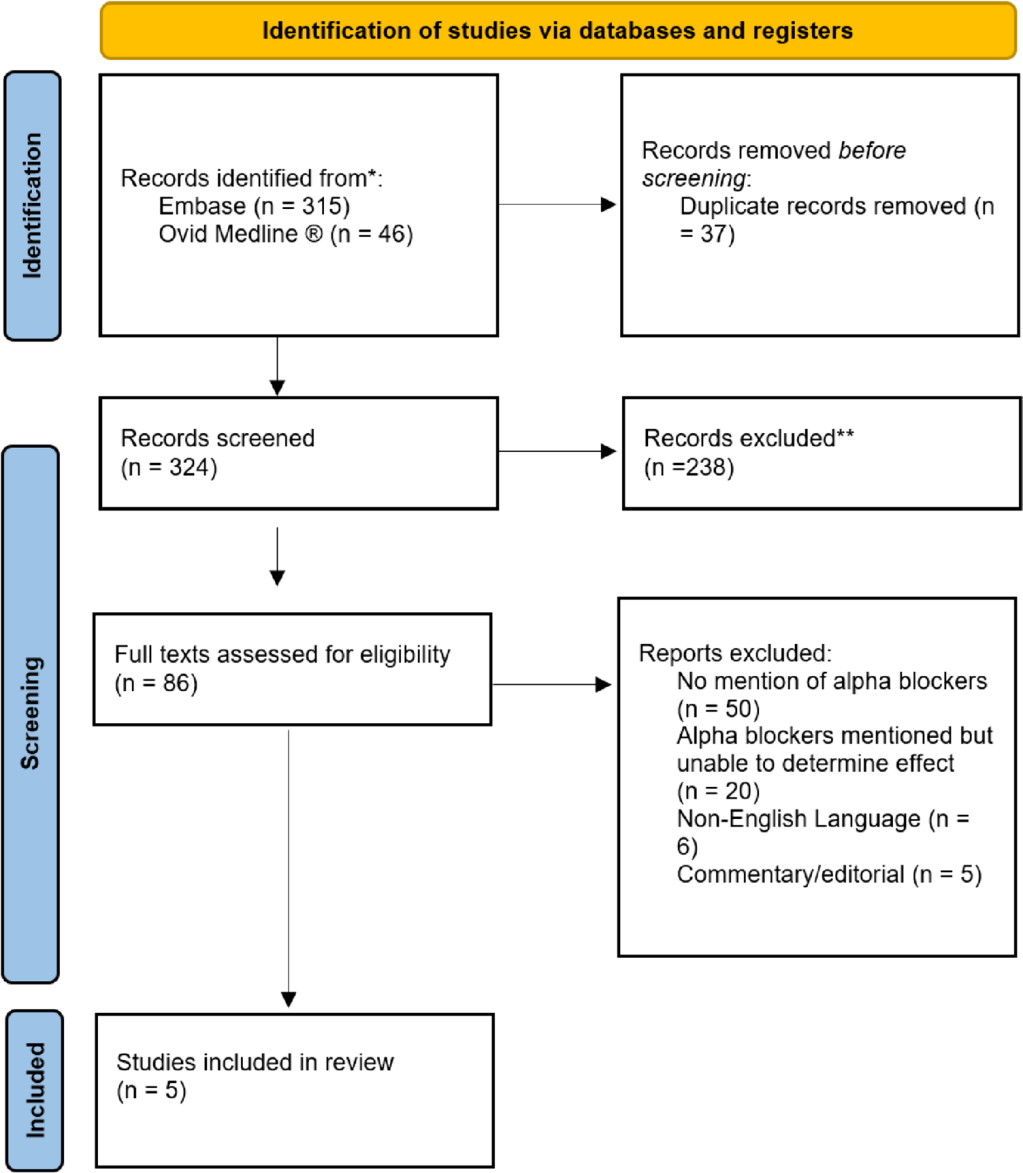
PRISMA diagram outlining the search strategy. 324 records were identified from Embase and Ovid Medline after accounting for duplicates. 238 records were removed at the initial title and abstract screening with a further 86 at full-text screening, leaving 5 records included in the review

The literature searches yielded 361 articles (315 from Embase and 46 from Medline). The online tool Covidence matched 37 records as duplicates and removed them from the pool resulting in 324 records entering the initial screening. Of these 324, 86 texts were identified as potentially fulfilling the inclusion and exclusion criteria and underwent full-text screening. Of these 86, 50 were excluded due to no mention of alpha-blocker usage in their methods, and 20 were excluded as there was either a missing comparator or lack of data that prevented the effect of alpha-blocker usage from being established. Six were removed due to the original article being in a non-English language with no available translation, and five were excluded due to their editorial nature. This left five studies to be evaluated as fulfilling the inclusion and exclusion criteria. A flow diagram using the PRISMA template is presented in Fig. 1.

### Characteristics of included studies

Five studies met the literature search criteria and were eligible for inclusion. Table 1, Table 2, Table 3 is an overview of the type of each study, its aims and the alpha-blocker protocol utilised.

**Table 1:** Overview of included studies

Author	Year	Type of study	Centre type	Study aims	Alpha blocker protocol
Muthuveloe et al	2016	Prospective Cohort	Single tertiary centre	To present transperineal template-guided prostate biopsy (template biopsy) outcomes at a tertiary referral centre	Single 400 μg tamsulosin perioperative dose
Kum et al	2019	Retrospective Data Collection	Single tertiary centre	To determine patient and procedure-related factors, which influence the occurrence of urinary retention after transperineal template biopsy	Single 400 μg tamsulosin perioperative dose
Loeb et al	2013	Systematic Review	—	To perform a systematic review of complications from prostate biopsy	—
Ekwueme et al	2013	Prospective Cohort	Single tertiary centre	To determine the incidence of prostate cancer, and pathological grade and location of prostate cancer, using a modified transperineal template-guided saturation biopsy To compare the acute urinary retention (AUR) rate found using modified TTSB with that of published reports	Patients continuing long-term tamsulosin
Namekawa et al	2015	Prospective Cohort	Single tertiary centre	To assess the adverse events associated with transrectal ultrasound guided TP 16-core prostate biopsy at a single institution	Patients continuing long term alpha blocker

Three prospective cohort studies, a single retrospective data collection and a single systematic review were identified through the search. Two studies had their patients take a single 400 μg tamsulosin dose whilst two studies had their patients continue their long-term alpha blockers

**Table 2:** Overview of study methods

Author	Year	Biopsy Technique	Anaesthetic	Median number of cores (range)	Data Collection method	Participants
Muthuveloe et al	2016	5 mm Brachytherapy grid template guided	GA	24 (24–28)	Case notes and database review	200
Kum et al	2019	5 mm Brachytherapy grid template guided + plus additional cores MRI as needed	GA or Regional	33 (10–54)	Retrospective data collection analysis	243
Ekwueme et al	2013	5 mm Brachytherapy grid template guided avoiding peri-urethral area	GA	28 (16–43)	Not mentioned	270
Namekawa et al	2015	Systematic 16 core biopsy	Regional	16 (16)	Daily questionnaires for 7 days postop	1663

Two studies used a template grid technique, one study used a modified template grid technique, one study used a 16-core systematic technique

**Table 3:** Overview of study results

Author	Year	α –blocker participant (% of total participants)	Patients on a-blocker at baseline (% of total participants)	Patients given alpha blocker peri-procedurally (% of total participants)	Non α– blocker participants (% of total participants)	α -blocker AUR Rate (number)	Non α – blocker AUR Rate (number)
Muthuveloe et al	2016	59/200 (29.5%)	Not applicable	59/200 (29.5%)	141/200 (60.5%)	5.30% (4)	12.50% (21)
Kum et al	2019	238/243 (97.9%)	56/243 (23.0%)	238/243 (97.9%) were given an alpha blocker on day of procedure (did not specify who the 5 participants were who did not receive therapy), and if not on alpha blocker at baseline were given a 5–7 day course post-operatively	5/243 (2.0%)	12.80% (5)	0% (0)
Ekwueme et al	2013	43/270 (16.0%)	43/270 (16.0%)	Not applicable	227/270 (84.0%)	9.30% (4)	4.40% (10)
Namekawa et al	2015	566/1663 (33.4%)	566/1663 (33.4%)	Not applicable	1097/1663 (66.6%)	19.61% (111)	12.22% (134)

One study demonstrated a reduction in AUR following prophylactic alpha blockers whilst three studies demonstrated a harm effect. Kum et al. did not have a case of urinary retention in their non-alpha blocker participants

#### Design and participants

Three of the five studies were of a prospective cohort study design at a single tertiary centre [12–14]. Kum et al. [15] was a retrospective data collection at a single tertiary centre, and Loeb et al. [16] was a systematic review examining the complications of prostate biopsy.

Of the prospective and retrospective studies, participants were all males suspected of prostate cancer undergoing either surveillance or diagnostic biopsies.

#### Biopsy technique and alpha-blocker regimens

The biopsy technique varied across the different studies. Muthuveloe et al. [12] used a transperineal template-guided approach, taking systematic biopsies with a 5 mm brachytherapy grid and a minimum of 24 cores. Kum et al. [15] used a similar technique while collecting additional targeted biopsy cores based on MP-MRI results when required. Namekawa et al. [14] used a predetermined 16-core systematic biopsy technique. Ekwueme et al. [13] explored a modified template-guided technique, using 22 mm long cores to avoid sampling the base of the peri-urethral area. They achieved this due to the length of the cephalo-caudal prostate increasing as the sampling moves centrally, hypothesising that reducing trauma to this region would reduce the AUR complication rates. There were no studies that met the criteria examining a freehand technique.

In terms of peri-operative alpha blocker regimes, two studies prescribed tamsulosin. Kum et al. [15] used 400 μg on the day of the procedure (given to 238/243 patients) followed by a five to seven-day course post-operatively given to all patients who were not initially on alpha-blocker therapy, whilst Muthuveloe et al. [12] only gave a single 400 μg dose of tamsulosin on the day of the procedure to 59 out of their 200 patients.

Two studies did not prescribe a prophylactic alpha-blocker on the day of or following the procedure. Ekwueme et al. [13] however, did report that 43 of their patients were on a long-term dose of 400 μg daily tamsulosin, including on the day of the procedure. Namekawa et al. [14] reported that 566/1663 had a history of alpha-blocker use but did not give a further breakdown of the exact medications and their usage during the perioperative period.

#### Data collection methods

The three prospective cohort studies all used similar methods to collect their data. Muthuveloe et al. [12] recruited their initial 200 participants and then retrospectively reviewed their case notes and databases. Namekawa et al. [14] delivered purpose-built daily questionnaires for 7 days following biopsy to their 2086 initially identified patients. The questionnaire focused on adverse events experienced after the biopsy. They received valid responses from 1663 patients which then underwent further analysis. Ekwueme et al. [13] did not describe how they collected their data on their prospective cohort other than to say their local audit committee approved it.

The systematic review by Loeb et al. [16] used the search terms Prostate Biopsy AND Complications to search PubMed and Embase retrieving 4402 records to be screened that were then narrowed down to 213 studies included in the final synthesis. 25 of these studies examined the morbidity following TPB.

#### Urinary retention rates

Two studies reported a potential benefit of using of an alpha-blocker in the perioperative period. Muthuveloe et al. [12] gave 59 of their 200 patients prophylactic tamsulosin and reported that the AUR rate dropped from 14.9% to 5.3% with no significant difference between the baseline characteristics of the two groups.

In their systematic review, Loeb et al. [16] found that the range of AUR varied across 24 studies examining TPB to be between 1.6 and 8.8%, with a single 25th outlier study at 20.6%. They concluded that this increase could be due to the lack of routine perioperative alpha-blockers, which was standard in the other 24 studies.

The two studies that only reported a history of, or current alpha-blocker usage demonstrated potential harm for perioperative tamsulosin on AUR rates. In the study by Ekwueme et al. [13], the patients on long-term 400 μg tamsulosin experienced urinary retention at a rate of 9.3% compared to 4.4% of those not taking an alpha-blocker. Similarly, Namekawa et al. [14] found a urinary retention rate of 19.6% compared to 12.2% in those taking alpha-blockers versus those that were not respectively.

The last study by Kum et al. [15] similarly reported that the rates of urinary retention were higher in the perioperative tamsulosin group at 13.0% compared to 0%; however, only 5 out of the 243 patients did not receive perioperative tamsulosin.

## Discussion

The shift away from TRUSB in recent years has largely been driven by the desire to reduce the risk of infective complications and sepsis. A national database review of 486,467 prostate biopsies in the United Kingdom National Health Service (NHS) found that the rates of sepsis had more than doubled in the two years to 2019 compared to the entire decade at 1.12% [17]. This has primarily been thought to be due to emerging fluoroquinolone resistance that has been confirmed in multiple studies and a systematic review prompting a shift towards TPB in the hope of reducing the burden of infective complications [2, 16, 18–20]. This shift has significantly dropped the morbidity related to sepsis with the procedure, however, AUR is now the leading complication after biopsy [21]. Perioperative tamsulosin is thought to reduce the rates of urinary retention through its uroselective alpha blockade mechanism.

Five studies examined the effect of the perioperative use of alpha blockers and the impact on AUR rates.

### Evidence for alpha blockers preventing AUR in TPB

Muthuveloe et al. [12] in their small prospective cohort series of 200 participants, reported AUR rates dropped from 12.5% to 5.3% when perioperative tamsulosin was used as part of their biopsy procedure in 59 of 200 participants, concluding there is a benefit for their usage in TPB. Measuring the effect of alpha blockers was not a direct aim of their observational study however, and there were no formal treatment groups or randomisation to determine the prescription of alpha blockers leading to a risk of selection bias. The baseline demographics for the study were given which included age, median PSA and pre-procedural PIRADS score on MRI, however, the authors did not provide a breakdown for their alpha blocker and non-alpha blocker groups, only stating that they were not different at baseline. There were no mentions of prostate volume or long-term alpha-blocker use in the study design, which other authors have suggested may increase the likelihood of urinary retention [13, 14, 16]. This leads to a risk of confounding bias as there was no mention of controlling for these factors.

In their systematic review, Loeb et al. [16] also concluded that alpha blockers may be beneficial in reducing AUR in TPB. They examined 25 studies that reported on the complications following TPB as part of their study, noting that AUR rates were between 1.6% and 8.8% for 24 of the studies where alpha blockers were given in the perioperative period, with a single outlier study at 20.6% not using alpha-blockers. The limitations of this conclusion however are significant, given the studies were all performed with differing biopsy techniques, surgeons, and study populations, making a single outlier study not an unexpected finding, but still worth commenting on as part of this review given the paucity of evidence.

### Evidence against alpha blockers preventing AUR in TPB

Three studies identified in this review reported a potential harmful effect for alpha blockers in reducing AUR.

In their small retrospective data analysis, Kum et al. [15] reported that their AUR was 12.8% for those taking alpha-blockers, compared to 0% for those that were not. This study was severely limited by its participant numbers and statistical power, with only 5/243 patients observed to be not taking alpha blockers in the perioperative period. AUR rates have been reported to be as low as 1–2% following biopsy, and a larger population than five would be required to power the study significantly enough to make a comment on the potential impact of alpha blockers, cautioning any conclusions drawn regarding their use here due to a potential selection bias.

Likewise, the remaining two studies in this review demonstrating the harmful effect for alpha blockers are significantly limited. Ekwueme et al. [13] and Namekawa et al. [14] reported their AUR rates increased by 111% and 61%, respectively, following the addition of alpha blockers. However, their study had patients already prescribed long-term alpha blockers before the biopsy continuing them in the perioperative period. As a result, there is a serious risk for selection and confounding bias, as these patients are more likely to enter retention at baseline given their indication for long-term alpha blocker prescription. This selection bias severely limits the conclusions that can be drawn from these two observational studies regarding prophylactic use in the perioperative period only, and the harm effect can be well explained in this setting. It is unlikely that the alpha-blockers were causing the observed increase in AUR rates.

### Limitations and gaps in the literature

There remains a significant gap in the literature concerning the prophylactic use of alpha blockers to prevent AUR following TPB. According to the literature search, there have been no RCTs examining this subject. Neither have there been any observational studies directly assessing this question. The data extracted in this review is drawn from the minor findings of studies outside of their aims and thereby is susceptible to selection and information bias. Our conclusions are based on data that has been collected without methods designed to specifically limit the number of potential confounding factors and improve the validity of the results regarding the effects of alpha blockers. Randomised controlled trial comparisons are needed in this field to accurately represent this risk and reduce the risk of selection and information bias.

In addition to this, much of the observational literature around TPB already has the entire population taking perioperative alpha-blockers based on the evidence from TRUSB, meaning the potential benefit or harm cannot be assessed in that population without a control comparator. There is significant scope for an RCT to be developed to fill this gap in the literature, and to provide an evidence base to continue this practice that it appears has solely been extrapolated from TRUSB.

### Predicting AUR retention risk

One study included in this review focused on assessing the risk factors for AUR following TPB. Kum et al. [15] concluded that the factors associated with an increased risk of urinary retention were patients of advanced age (> 68.7 years); those with a larger prostate volume (> 75 cc; a higher number of biopsy cores taken (> 35) and a higher international prostate symptom score (IPSS) before biopsy. These conclusions are reflected in the larger literature, with several studies also finding that a greater number of core biopsies, larger prostatic volume and older age are independent risk factors for entering AUR [4, 22–24]. Patients also on long term alpha blockers are more likely to enter retention. [13, 14]. As such, this population of patients are the most likely to benefit from alpha-blockers in terms of risk stratification and should, at the very least, be considered for a prophylactic perioperative regime.

Another early identified risk factor associated with developing AUR following TPB was the use of muscle relaxants as part of the general anaesthetic due to their anti-muscarinic effects [25]. Subsequently, it was recommended that paralytics be avoided in the procedure [25], and they have largely been phased out of practice [26].

### Alpha-blocker protocol

There is no standard clinical protocol or guideline for prophylactic alpha-blockers. Muthuveloe et al. [12] used a single dose of 400 μg tamsulosin the day of the procedure to achieve their reduction in AUR rates and the published literature reports similar varying practices from single doses of 400 μg to 2-week courses of 800 μg starting two prior to biopsy [15]. Further research is required to determine the optimum protocol.

## Conclusion

AUR remains the leading complication after TPB. This review found a significant gap in the literature regarding the evidence base for perioperative alpha-blockers to prevent AUR following TPB, however there is some weak evidence from observational studies that there may be benefit. The scope and need for an RCT remains. There is no current evidence-based alpha-blocker protocol to prevent retention and clinical practice varies significantly in terms of their usage. In terms of triaging risk, older patients with larger prostatic volumes undergoing a high number of core biopsies appear to be most at risk of entering retention and would therefore benefit the most from the use of perioperative alpha-blockers.

## Data Availability

The authors confirm that the data supporting the findings of this study are available within the article.

## Appendix

### Appendix 1 Search strategy

**Table:**

Ovid Medline (R) (accessed on 15/11/2023)
Search strategy:
1 Transperineal Biopsy.mp. (3140)
2 Urinary Retention\ (5009)
3 urine retention.mp. (699)
4 2 OR 3 (5546)
5 1 AND 4 (46)
Embase classic + Embase (accessed on 15/11/2023)
Search strategy:
1 Transperineal.mp. (6476)
2 urinary retention.mp. (19708)
3 urine retention/. (36035)
4 2 OR 3 (39551)
5 1 AND 4 (570)
6 Limit 5 to article or “review” (315)

### Appendix 2 Inclusion and exclusion criteria

**Table:**

Inclusion criteria	Exclusion criteria
RCT, Retrospective studies, prospective cohort studies, national database series, systematic reviews/meta-analysis Study examining TPB with a proportion of patients taking at least one alpha blocker in the perioperative period Data provided on the number of patients taking alpha blockers and those not Data provided on what number of alpha blocker recipients entered AUR and what number of non- alpha blocker recipients entered AUR	Non-English Language Narrative/editorial review only Full text not available/abstract only Methods did not mention the perioperative usage of alpha blockers Reported AUR complication rates did not provide a breakdown on participant usage of alpha blockers

## Abbreviations

TPB: Transperineal Prostate Biopsy
TRUSB: Transrectal Ultrasound Guided Prostate Biopsy
AUR: Acute Urinary Retention
RCT: Randomised Controlled Trial
NHS: National Health Service

## References

[1] Leslie, S.W., et al., *Prostate Cancer*, in *StatPearls*. 2024, StatPearls Publishing Copyright © 2024, StatPearls Publishing LLC.: Treasure Island (FL).

[2] Borghesi M et al (2017) Complications after systematic, random, and image-guided prostate biopsy. Eur Urol 71(3):353–365 27543165 10.1016/j.eururo.2016.08.004

[3] Rafiq M et al (2022) Sepsis rates after template prostate biopsy with single-dose prophylactic antibiotic. Cent European J Urol 75(2):205–208 35937653 10.5173/ceju.2022.0229 PMC9326692

[4] Kohl T et al (2022) Comprehensive analysis of complications after transperineal prostate biopsy without antibiotic prophylaxis: results of a multicenter trial with 30 days’ follow-up. Prostate Cancer Prostatic Dis 25(2):264–268 34267332 10.1038/s41391-021-00423-3 PMC9184280

[5] Stefanova V et al (2019) Transperineal prostate biopsies using local anesthesia: experience with 1,287 prostate cancer detection rate, complications and patient tolerability. J Urol 201(6):1121–1126 30835607 10.1097/JU.0000000000000156

[6] Thomson A et al (2020) Transperineal prostate biopsy: a review of technique. Transl Androl Urol 9(6):3009–3017 33457274 10.21037/tau.2019.12.40 PMC7807331

[7] Bozlu M et al (2003) Voiding impairment after prostate biopsy: does tamsulosin treatment before biopsy decrease this morbidity? Urology 62(6):1050–1053 14665353 10.1016/j.urology.2003.07.006

[8] Chung SJ et al (2015) The preventive effect of tamsulosin on voiding dysfunction after prostate biopsy: a prospective, open-label, observational study. Int Urol Nephrol 47(5):711–715 25812823 10.1007/s11255-015-0955-7

[9] Dunn CJ, Matheson A, Faulds DM (2002) Tamsulosin: a review of its pharmacology and therapeutic efficacy in the management of lower urinary tract symptoms. Drugs Aging 19(2):135–161 11950378 10.2165/00002512-200219020-00004

[10] Covidence systematic review software, Veritas Health Innovation, Melbourne, Australia. Available at www.covidence.org.

[11] National Heart, Lung, and Blood Institute. "Study Quality Assessment Tools [https://www.nhlbi.nih.gov/health-topics/study-quality-assessment-tools]." (2019).

[12] Muthuveloe D et al (2016) The detection and upgrade rates of prostate adenocarcinoma following transperineal template-guided prostate biopsy - a tertiary referral centre experience. Cent European J Urol 69(1):42–47 27123325 10.5173/ceju.2016.675 PMC4846721

[13] Ekwueme K et al (2013) Transperineal template-guided saturation biopsy using a modified technique: outcome of 270 cases requiring repeat prostate biopsy. BJU Int 111(8):E365–E373 23714648 10.1111/bju.12134

[14] Namekawa T et al (2015) Prospective evaluation of the safety of transrectal ultrasound-guided transperineal prostate biopsy based on adverse events. Int J Clin Oncol 20(6):1185–1191 25917775 10.1007/s10147-015-0831-6

[15] Kum F, Jones A, Nigam R (2019) Factors influencing urinary retention after transperineal template biopsy of the prostate: outcomes from a regional cancer centre. World J Urol 37(2):337–342 29974188 10.1007/s00345-018-2390-8

[16] Loeb S et al (2013) Systematic review of complications of prostate biopsy. Eur Urol 64(6):876–892 23787356 10.1016/j.eururo.2013.05.049

[17] Tamhankar AS et al (2020) The clinical and financial implications of a decade of prostate biopsies in the NHS: analysis of Hospital Episode Statistics data 2008–2019. BJU Int 126(1):133–141 32232966 10.1111/bju.15062

[18] Zaytoun OM et al (2011) Emergence of fluoroquinolone-resistant Escherichia coli as cause of postprostate biopsy infection: implications for prophylaxis and treatment. Urology 77(5):1035–1041 21420152 10.1016/j.urology.2010.12.067

[19] Carignan A et al (2012) Increasing risk of infectious complications after transrectal ultrasound-guided prostate biopsies: time to reassess antimicrobial prophylaxis? Eur Urol 62(3):453–459 22575912 10.1016/j.eururo.2012.04.044

[20] Wagenlehner FM et al (2013) Infective complications after prostate biopsy: outcome of the Global Prevalence Study of Infections in Urology (GPIU) 2010 and 2011, a prospective multinational multicentre prostate biopsy study. Eur Urol 63(3):521–527 22704727 10.1016/j.eururo.2012.06.003

[21] Devetzis K, Kum F, Popert R (2021) Recent advances in systematic and targeted prostate biopsies. Res Rep Urol 13:799–809 34805013 10.2147/RRU.S291963 PMC8598205

[22] Skouteris V, Stone N (2022) MP09-05 comprehensive analysis of morbidity following transperineal prostate biopsy. J Urol 207(Supplement 5):e136

[23] Pepe P, Aragona F (2014) Prostate biopsy: results and advantages of the transperineal approach–twenty-year experience of a single center. World J Urol 32(2):373–377 23743734 10.1007/s00345-013-1108-1

[24] Pepe P, Aragona F (2013) Morbidity after transperineal prostate biopsy in 3000 patients undergoing 12 vs 18 vs more than 24 needle cores. Urology 81(6):1142–1146 23726443 10.1016/j.urology.2013.02.019

[25] Willis S, Bott S, Montgomery B (2013) Urinary retention following transperineal template prostate biopsy – study of risk factors. J Clic Urology 6(1):55–58

[26] Berry B et al (2020) Comparison of complications after transrectal and transperineal prostate biopsy: a national population-based study. BJU Int 126(1):97–103 32124525 10.1111/bju.15039

